# Macrophage Polarization: Learning to Manage It

**DOI:** 10.3390/ijms23137208

**Published:** 2022-06-29

**Authors:** Nadia Lampiasi

**Affiliations:** Consiglio Nazionale delle Ricerche, Istituto per la Ricerca e l’Innovazione Biomedica, Via Ugo La Malfa 153, 90146 Palermo, Italy; nadia.lampiasi@irib.cnr.it; Tel.: +39-09-1680-9513; Fax: +39-09-1689-5548

To date, four reviews and seven experimental articles have been published in this Special Issue. The common thread of this Special Issue is the polarization of macrophages and their involvement in both homeostatic and pathological conditions. The reviews published cover topics concerning the involvement of macrophages in the most common inflammatory diseases, including the function they perform with other innate immune cells (e.g. mast cells) in the female reproductive system and oncological diseases. Furthermore, through experimental articles we have seen that macrophage polarization is implicated in many different pathologies (diabetes, atherosclerosis) and in osteoclastogenesis, as well as being useful for immunotherapies in the treatment of cancer by increasing macrophage immune reactivity.

Tissue resident macrophages (Mϕs) are very plastic phagocytic cells, devoted to scavenging endogenous threats (senescent or transformed cells, dead cells, cellular debris, and misfolded/aggregated proteins) and which contribute to physiological remodeling and wound healing in homeostatic conditions. In many organs, (Mɸs) are derived from precursor cells of fetal origin that are self-renewing and long-lived, and maintain a homeostatic pool without the contribution of infiltrating monocytes. However, in some tissues monocyte-derived macrophages can be recruited, and they are derived from adult progenitors, have a shorter lifespan and, in some circumstances, can replace tissue-resident Mɸs. Embryonic tissue-resident and adulthood Mɸs from tissue-infiltrating monocytes play distinct roles, both in health and disease as well as in the female reproductive system [1], or in splenic red pulp, where Mϕs engulf erythrocytes contributing to recycling heme [2] (Figure 1A,B). 

In tissues, monocytes respond to environmental signals by acquiring distinct functional phenotypes. Indeed, macrophages undergo classical activation (M1) or alternative activation (M2) in response to various signals, even if a plethora of intermediate cellular phenotypes exist [3] (Figure 1C). The M1 phenotype is usually activated through TLR and IFN-γ stimulation, whereas the M2 phenotype is activated through IL-4 and IL-13 cytokines stimulation. M1 is a killing phenotype and is devoted to the production of inflammatory molecules and cytokines such as TNF-α, IL-1β, IL-6, IL-12 and IFN-γ, and toxic molecules such as reactive oxygen species (ROS), nitric oxide (NO) and antimicrobial peptides. M2 is a repair phenotype, and is characterized by increased production of IL10, IL-4R and VEGF, as well as low levels of pro-inflammatory molecules, since its function is the dampening of Type 1 inflammation and promoting wound-healing (Figure 1C) [1,2,3].

An important example of tissue-specific adaptation of Mϕs is represented by osteoclasts (OCs), large, multinucleated cells that participate in physiological bone metabolism by resorbing bone in coordination with osteoblasts, which generate new bone. One paper of this Special Issue deals with the subject of “biomaterial inflammasome” [5] (Figure 2A).

Barbeck et al. investigated the monocyte–macrophage cellular response to biomaterial used for bone implantation [5]. The main result was that the pore size, the granule shape and the diameter of the biomaterial used have an influence on the degradation of the material mediated by macrophages, and these factors were important for the functionality in correlation with the process of osteoconductivity [5]. Another paper investigated the role of p38 MAPK signaling in macrophages during bone fracture healing [6]. The mechanical vibration used in this study to facilitate fracture repair did not affect the polarization from M1 to M2, but instead promoted differentiation into a specific subset of dendritic monocytes/macrophages with the ability to produce both inflammatory and anti-inflammatory cytokines, suggesting that this cellular subtype does not fit a conventional M1/M2 classification [6] (Figure 2B). 

Remarkably, Mattorre and colleagues, for the first time, reported the existence of a short form of aminopeptidase ERAP2 secreted exclusively by macrophages of the M2-subtype in all individuals regardless of the haplotype expressing the full-length form of ERAP2 [7]. It should be noted that the binding of ERAP2 to IRAP (called oxytocinase) can interfere with the renin-angiotensin system (RAS) by inhibiting its anti-inflammatory properties, and thus contributing to many pathologies ranging from autoimmune and inflammatory diseases, hypertension and cancer to SARS-CoV-2 [7] (Figure 2C).

Depending on the microenviromental signals and the local macrophage subtype polarization, the inflammation can become chronic, and the homeostatic conditions are not restored. Chronic inflammation is a key feature of the tumor microenvironment, not only stimulating proliferation and survival of tumor cells, but also suppressing antitumor immunity. Monocytes from the blood are recruited to the tumor microenvironment and become tumor-associated macrophages (TAMs), playing a role in the development of tumor cells and in immune evasion or in immunosuppression [4] (Figure 1D). Indeed, activated Mϕs (M1-subtype) can defend against tumors by directing tumor cytotoxicity and by secreting cytokines, whereas an increased number of M2-phenotype is associated with poor prognosis as, for example, in primary breast cancer. One interesting paper in the Special Issue focused on the use of heat-killing preparation of *Mycobacterium obuense* (*M. obuense*) as an adjunctive immunotherapeutic agent for the treatment of cancer, since it may trigger the differentiation of human monocytes into a monocyte-derived macrophage (MDM) with the potential to induce the antitumor activity of macrophages [8] (Figure 2D). Another paper of the Special Issue investigated the ameliorative effects of protease inhibitors (PI) extracted from *Avena sativa (A. sativa)* on dampening chronic inflammation contributing to the development of a tolerogenic phenotype of macrophages [9] (Figure 2E). Among the diseases sustained by chronic inflammation there are atherosclerosis and type 2 diabetes (T2D). Hypercholesterolemia is one of the underlying conditions of atherosclerotic plaques and statins are today the most prescribed class of drugs worldwide to contrast hypercholesterolemia through their antioxidant or anti-inflammatory effect. The paper of Linnenberger and colleagues compared the pro- and anti-inflammatory effects of statins with bempedoic acid (BA), recently registered as a drug for the treatment of hypercholesterolemia. They concluded that BA is effective in the rescue of the phagocytic activity of macrophages differently from statins, and does not induce side effects as statins do [10] (Figure 2F). Modulation of macrophage phenotypes has been suggested as a novel strategy for the treatment of inflammatory pathologies. The paper of Abidov and colleagues published in the Special Issue addressed T2D pathology with an in vivo study [11] (Figure 2G). Chronic inflammation, generated by T2D progression, increases tissue damage with the involvement of macrophage-produced mediators in both tissue injury and tissue repair. This study investigated the effect of sodium aminophthalhydrazide (APH) on the generation of extra-islet insulin-producing cells (IPC) in acini and pancreatic ducts in rats with or without diabetes. The results indicated that APH modulates the functional activity of macrophages, shifting them to a reparative anti-inflammatory phenotype and decreasing macrophage infiltration and inflammation in the pancreas [11]. 

Overall, this Special Issue has covered various topics of inflammatory conditions which depend on macrophage polarization. I hope this Special Issue attracts the attention and interest of the scientific community and inspires researchers to continue the exploration of the scientific topics addressed here.

## Figures and Tables

**Figure 1:**
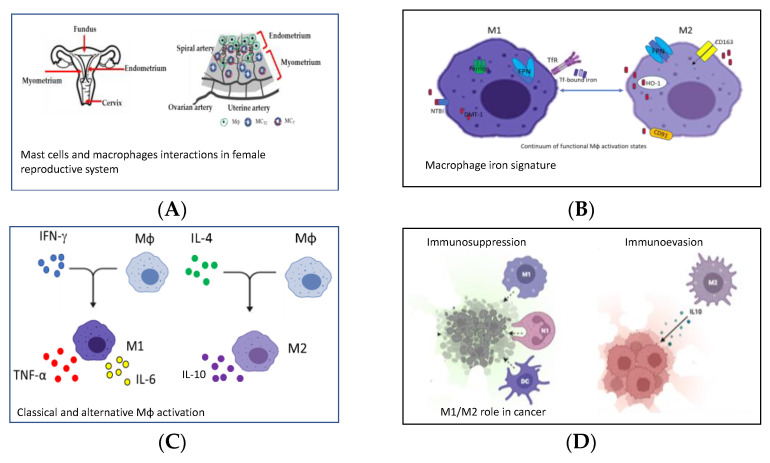
Representative pictures of the four reviews published in the Special Issue. This figure was generated with BioRender and PowerPoint. (**A**) Mast cells and macrophages interactions in emale Reproductive System [1]. (**B**) Macrophage iron signature [2]. (**C**) Classical and alternative Mϕ activation [3]. (**D**) M1/M2 role in cancer [4].

**Figure 2:**
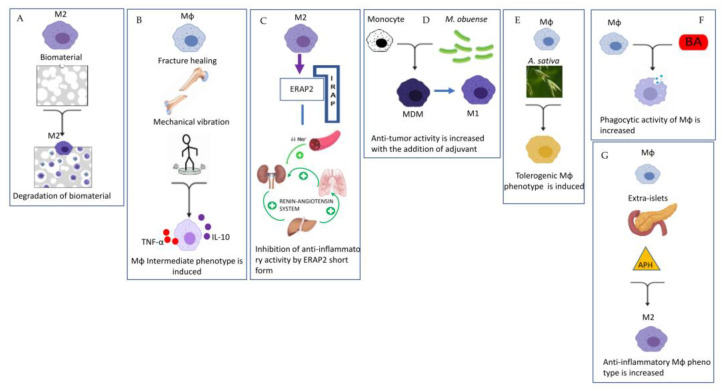
Representative pictures of the seven experimental papers published in the Special Issue. This figure was generated with BioRender and PowerPoint. (**A**) Degradation of biomaterial by Mϕ depends on the consistency of the biomaterial itself [5]. (**B**) Mechanical vibration induced an intermediate Mϕ phenotype in fracture healing [6]. (**C**) ERAP2 short form caused an inhibition of anti-inflammatory ativity of Renin Angiotensin System [7]. (**D**)Anti-tumor ativity is increased with the addition of adjuvant *M. obuense* [8]. (**E**) *A. sativa* induced a tolerogenic Mϕ phenotype [9]. (**F**) Bempedoic acid (BA) increased phagocytic activity of Mϕ [10]. (**G**) Sodium aminophthalhydrazide (APH) increased anti-inflammatory phenotype in the pancreas [11].
